# Science, democracy and emerging threats to scientific progress

**DOI:** 10.1186/1478-811X-10-24

**Published:** 2012-08-23

**Authors:** Stephan M Feller

**Affiliations:** 1 Biological Systems Architecture Group, Weatherall Institute of Molecular Medicine, Department of Oncology, University of Oxford, John Radcliffe Hospital, Headley Way, Oxford OX3 9DS, UK

## Abstract

Can trustworthy science flourish in countries suffering from dictatorship? This is an increasingly relevant question. Many commercial publishers want to maximise their profits (as is to be expected in a capitalistic system) and are pushing into non-democratic countries with rapid economic growth like China. But how much can we trust the papers coming from countries with dictatorial regimes?

## 

At present the vast majority of scientifically leading countries are ruled by democratic systems, with the USA, the EU, Japan and Australia dominating research output and quality. But this may change rapidly, at least in terms of output.

Clearly, even in the USA, Europe etc. the current peer review systems is not fool-proof ( https://www.scienceexchange.com/reproducibility) [1,2] and article retractions due to fabrication of data are on the rise in a fiercely competitive environment created by an economically difficult climate [3].

But are matters much worse in countries where people get promoted based on party membership and non-scientific network connections rather then on the quality of their scientific output? Does it hurt scientific progress if governments are able to suppress unpleasant data on environmental issues, the population health effects of pollutants, and negative effects of rigid population restriction measures on mental health and crime? Is it bad for scientific progress if people are protected from punishment for scientific fraud and for stealing the data of colleagues because a family member is high up in the party hierarchy? Can we trust clinical studies from countries where patients have little or no means to ensure they get decent treatment and where hospitals are used for the cheap and uncontrolled testing of new drugs? Which areas of scientific research, if any, can remain unaffected by uncontrolled governmental intervention and/or lack of protection of human rights?

Scientific journal publishers should tread very carefully and must not ignore these issues.

If they do, scientists concerned about growing obstacles to scientific progress might take the publishing of scientific papers back into their own hands.

After all, these days it needs little more than some relatively simple (and widely available) computer software and a couple of servers to publish electronic science journals. In the era of the internet, scientific societies of sufficient size do not really need commercial publishers anymore to effectively disseminate research papers on a global scale, so scientific journal publishers have effectively declined in their significance from a necessity to a convenience.
